# Protein neddylation as a therapeutic target: challenges and opportunities

**DOI:** 10.1172/JCI206924

**Published:** 2026-08-03

**Authors:** Shizhen Zhang, Huiyin Lan, Yi Sun

**Affiliations:** 1 Cancer Institute and; 2 Department of Breast Surgery of the Second Affiliated Hospital, Zhejiang University School of Medicine, Hangzhou, China.; 3 Department of Radiation Oncology, Zhejiang Cancer Hospital, Hangzhou Institute of Medicine, Chinese Academy of Sciences, Zhejiang Key Laboratory of Particle Radiotherapy Equipment, Hangzhou, China.; 4 Institute of Translational Medicine, Zhejiang University School of Medicine, Hangzhou, China.; 5 Zhejiang University Cancer Center, Hangzhou, China.; 6 Research Center for Life Science and Human Health, Binjiang Institute of Zhejiang University, Hangzhou, China.; 7 State Key Laboratory of Transvascular Implantation Devices, Second Affiliated Hospital, Zhejiang University School of Medicine, Hangzhou, China.

## Abstract

Protein neddylation is an evolutionarily conserved posttranslational modification that conjugates NEDD8 to its substrate, catalyzed by an E1-activating enzyme, E2-conjugating enzyme, and E3 ligase. Neddylation is essential for cellular homeostasis, and its dysregulation has been implicated in diverse human diseases, including cancer, neurodegenerative diseases, and metabolic disorders, making the process a promising therapeutic target. In this Review, we systematically summarize the biochemical activity and biological functions of neddylation; its alterations in human diseases, particularly in cancers; and its validation as an attractive target for cancer therapy. We provide an overview on the discovery of neddylation inhibitors and the progress of MLN4924 (pevonedistat) and TAS4464 clinical trials and critically evaluate the core challenges and emerging opportunities for therapeutic strategies targeting neddylation.

## Introduction

Neddylation is an evolutionarily conserved posttranslational modification that conjugates the ubiquitin-like protein neural precursor cell–expressed developmentally downregulated protein 8 (NEDD8) to target substrates (1). Neddylation is structurally and mechanistically similar to ubiquitylation, with both modifications employing a three-enzyme cascade of E1-activating, E2-conjugating, and E3 ligase enzymes. However, key differences exist: ubiquitylation primarily drives substrate for proteasomal degradation via K48-linked chains or alters substrate functions through the K63 linkage, whereas neddylation mainly alters the subcellular localization, stability, and function of its substrates (2, 3).

The neddylation modification begins with ATP-dependent activation of NEDD8 by the E1 enzyme Nedd8-activating enzyme (NAE, an NAE1-UBA3 heterodimer), forming a thioester bond. NEDD8 is then transferred to the catalytic cysteine of an E2 enzyme (UBE2M or UBE2F). Finally, an E3 ligase (e.g., RBX1 or RBX2) mediates the transfer of NEDD8 to a lysine residue on the substrates, which include cullins and noncullin proteins (4, 5). In addition, the scaffold-type E3 ligases defective in cullin neddylation 1–5 (DCN1–DCN5) cooperate with RBX1 or RBX2 to facilitate cullin neddylation (6).

**Figure 1:**
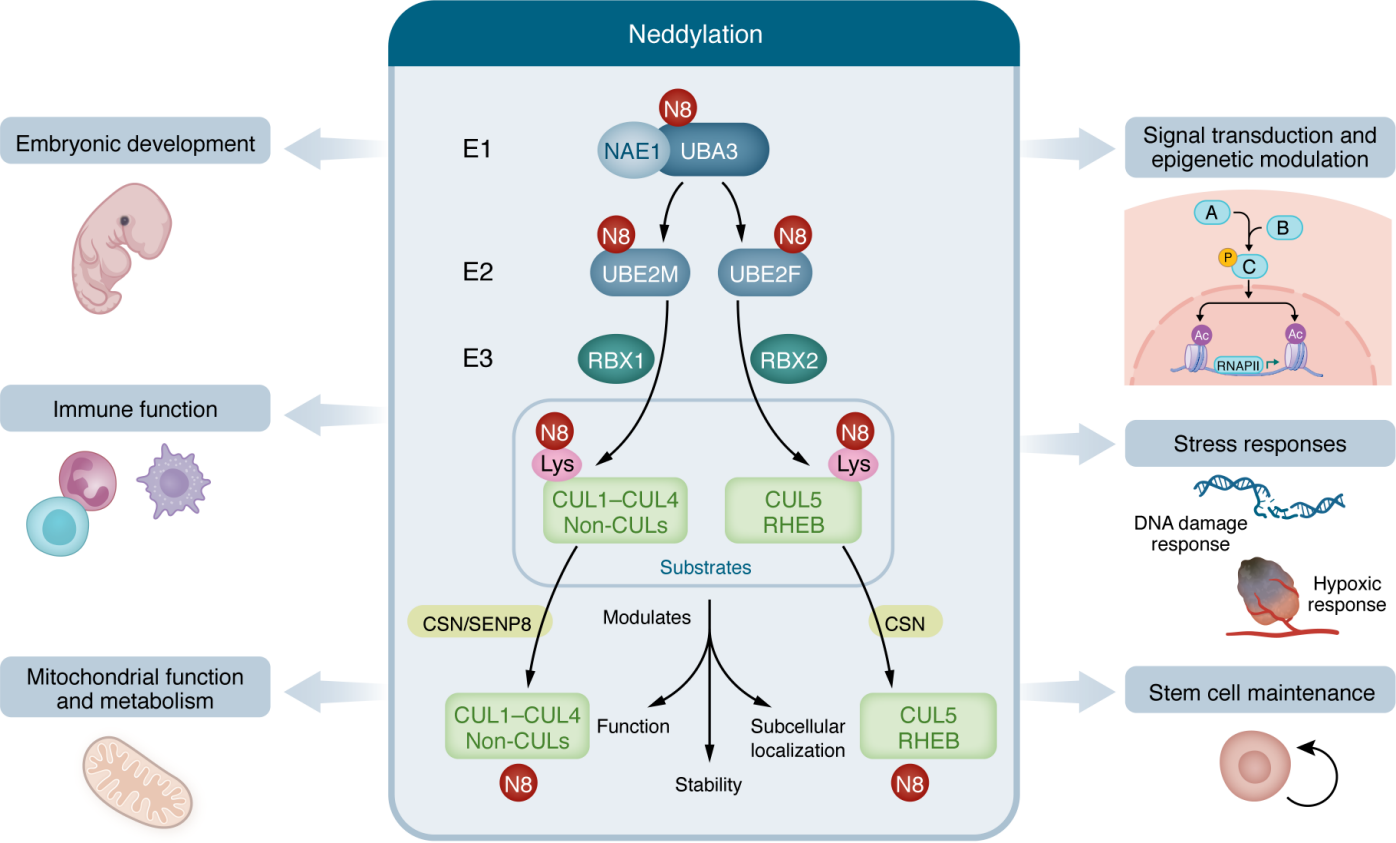
The neddylation modification and its physiological functions. The center of the figure shows the neddylation/deneddylation cascade, and images on the left and right show physiological functions regulated by neddylation.

The reverse process is deneddylation, which is catalyzed by deneddylases, including the COP9 signalosome (CSN), sentrin-specific protease 8 (SENP8, also known as DEN1 or NEDP1), ataxin-3, ubiquitin-specific peptidase 21 (USP21), and ubiquitin C-terminal hydrolase L3 (UCH-L3) (3, 4). The CSN complex is the major deneddylase, cleaving NEDD8 from both cullin and noncullin substrates (7). It binds tightly to deneddylated cullin-RING ligases (CRLs), sterically hindering the RBX1-E2 interaction to maintain CRLs in an inactive state. Substrate binding to the CRL competes with CSN, promoting reneddylation and CRL reactivation (8). Conversely, SENP8 shows high specificity for NEDD8 on noncullin proteins, acting as a complementary deneddylase to CSN (9, 10) (Figure 1, middle).

Cullin family proteins represent the best-characterized physiological substrates of neddylation. RBX1 couples with UBE2M to neddylate cullin-1–4 (CUL1–CUL4), whereas RBX2 couples with UBE2F to neddylate CUL5 (5). While CUL7 is not modified by neddylation under experimental conditions that induce robust neddylation of canonical cullins (11), a recent study showed that RBX1 and UBE2F neddylate CUL9 (12). Cullins are scaffold components of CRLs, the largest family of ubiquitin E3 ligases, and cullin neddylation induces conformational changes that activate CRLs to ubiquitylate approximately 20% of cellular proteins for proteasomal degradation (13, 14). The noncullin substrates of neddylation include oncogenes, tumor suppressors, ribosomal proteins, histones, transcription factors, and E3 ligases, underscoring the pleiotropic effects of neddylation (3, 4). Thus, by acting both directly on its substrates and indirectly through CRLs, neddylation is a master regulator of protein function and turnover, affecting embryonic development, immune function, mitochondrial function and energy metabolism, signal transduction, epigenetic modulation, stress responses, and stem cell maintenance, among other functions (13, 15) (Figure 1). Supplemental Tables 1 and 2 list all neddylation and deneddylation enzymes and their reported substrates (supplemental material available online with this article; https://doi.org/10.1172/JCI206924DS1).

Since the discovery of NEDD8 in 1992 (16), landmark studies have linked neddylation dysregulation to diverse pathologies (for reviews, see refs. 3, 17). The discovery of the first-in-class NAE inhibitor MLN4924 (pevonedistat) in 2009 (18) advanced neddylation research from basic biology to clinical trials. This Review focuses on the physiological functions of neddylation; its dysregulation in human diseases, particularly cancer; drug discovery efforts and clinical trials of neddylation inhibitors; and finally, the challenges and opportunities in the field of neddylation research.

## Physiological functions of neddylation

Neddylation orchestrates multiple essential biological processes to maintain homeostasis and normal physiology, including embryogenesis, immune function, metabolism, signal transduction, epigenetic modulation, stress responses, and stem cell maintenance (3, 19) (Figure 1).

### Embryonic development, organogenesis, and stem cell maintenance.

Neddylation is essential for mammalian development. Global knockout of *Uba3* causes embryonic lethality in mice (20). Similarly, knockout of either E3 ligase *Rbx1* or *Rbx2* results in embryonic lethality without mutual compensation, highlighting their nonredundant functions (21, 22). Tissue-specific knockout studies have shown critical roles for neddylation in neural development (22), neuronal migration (23, 24), cerebral cortex formation (25), postnatal brain cognitive function (26), and heart development (27). Neddylation also modulates stem cell homeostasis, self-renewal, and differentiation through distinct CRLs. For example, CRL1^FBXW7^ maintains embryonic and neural stem cell properties by degrading Notch and c-MYC (28). CRL1^β-TrCP^ inhibits differentiation of embryonic stem cells into neural stem cells by targeting RE1-silencing transcription factor (REST) for degradation (29). CRL2^VHL^ sustains hematopoietic stem cell quiescence through HIF-1α degradation (30), whereas CRL3^SPOP^ and CRL4^DCAF5^ suppress stem cell self-renewal by degrading NANOG and SOX2, respectively (31, 32). Finally, CRL5^ASB4^ promotes endothelial/trophoblast differentiation via ID2 degradation (33). Thus, by modulating the activation of these CRLs, neddylation is indispensable for early embryogenesis, organ formation, and precise control of stem cell populations.

### Immune function.

Neddylation modulates both innate and adaptive immunity. Briefly, neddylation has been shown to facilitate neutrophil recruitment in acute injury (34) and proinflammatory cytokine secretion by macrophages (35), but it differentially regulates inflammatory responses of neutrophils and macrophages (36) and affects DC function and survival (37–39). In adaptive immunity, neddylation regulates T cell proliferation, response, cytokine production, and maturation (40–42) and is required for the maintenance of Tregs (43).

### Mitochondrial function and metabolism.

Neddylation influences mitochondrial dynamics through CRL1^β-TrCP^-mediated mitofusin-1 degradation (44) and supports fatty acid β-oxidation by stabilizing the electron transport chain proteins ETFA and ETFB (45). Neddylation also plays a role in systemic metabolism. For example, phosphoenolpyruvate carboxykinase 1 neddylation enhances gluconeogenesis (46); neddylation stabilizes PPARγ and SREBP1c to regulate lipid metabolism (47–49); and UBE2M-mediated tripartite motif containing 21 (TRIM21) neddylation controls obesity-induced inflammation and metabolic disorders (50). Finally, neddylation activation of CRL3^SPOP^ and CRL3^KCTD10^ affects glutamine and cystine metabolism by targeting their transporters, ASCT2 and SLC7A11, respectively (51–53).

### Signal transduction and epigenetic modulation.

Neddylation modulates several key signaling pathways. These include NF-κB signaling, via CRL1-mediated IκB degradation or CRL5-mediated TRAF6 activation (34, 54), and RTK/MAPK signaling, in which c-Cbl–mediated neddylation stabilizes TGFβRII (55) but destabilizes EGFR (56). Neddylation also affects RAS/ERK signaling through UBE2M-mediated neddylation of SHC, promoting ERK activation (40). In addition, neddylation contributes to epigenetic regulation by affecting DNA methylation via the DNMT3b-CUL4A interaction (57) and histone modifications through CUL4A-DDB1–mediated methylation of histone H3 (58), and it negatively regulates H3K9me3 homeostasis through CRL5^ASB7^ (59).

### Stress responses.

Neddylation plays an essential role in cellular responses to stress conditions. For example, neddylation participates in response to hypoxia through CRL2^VHL^- or CRL5-mediated HIF-1α degradation (60, 61), to oxidative stress via CRL3^KEAP1^-mediated NRF2 degradation (62, 63), and to DNA damage and repair through the p53/MDM2 axis, DNA-PKcs activation, and histone H4 neddylation (64–66). During nucleolar stress, neddylation of RPL11 and RPS14 modulates p53 activity, helping cells adapt to ribosomal dysfunction (67–69).

## Pathological alteration of neddylation in cancer

**Figure 2:**
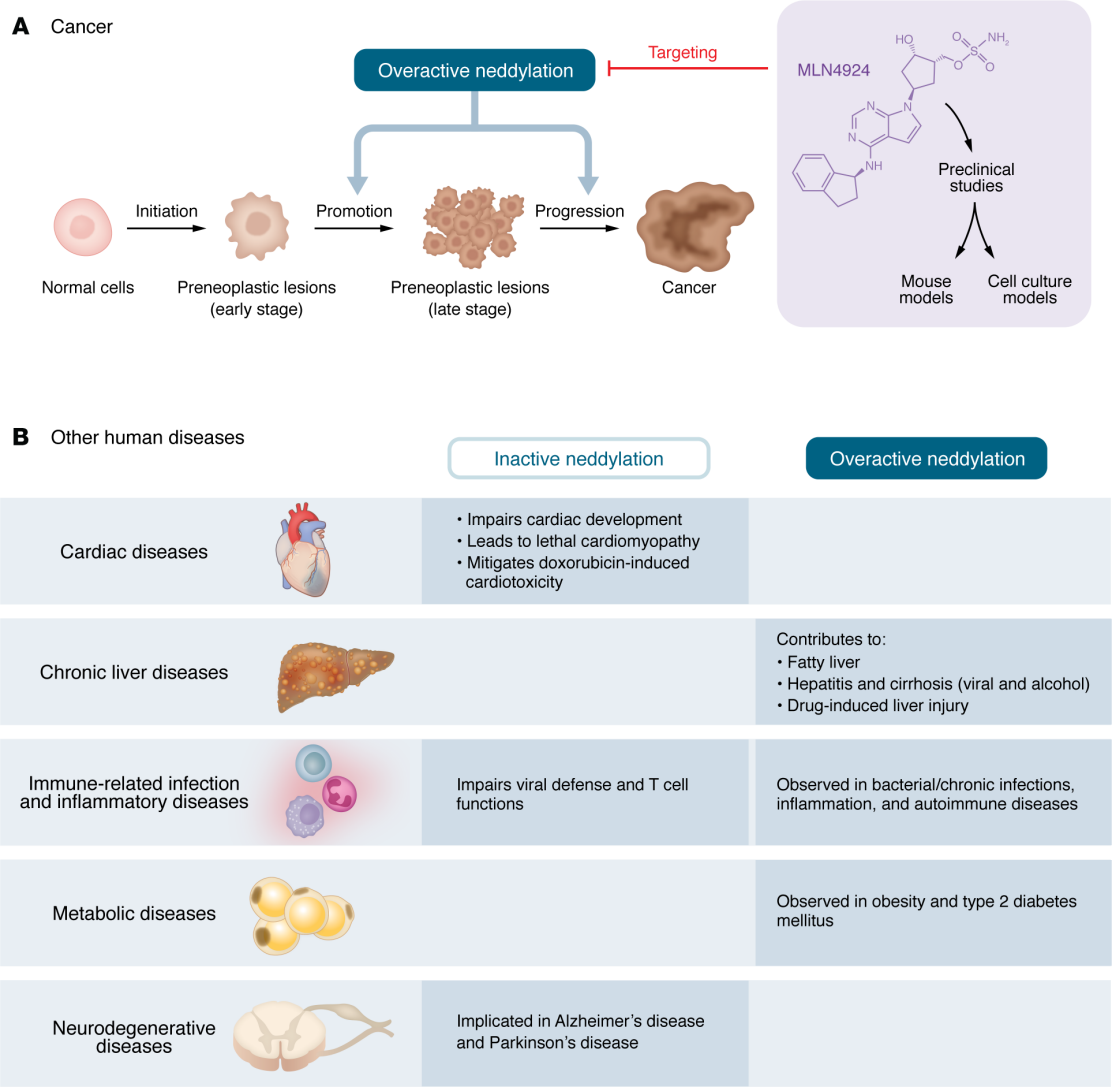
Dysregulated neddylation modification in human diseases. (**A**) Cancer: Overactivation of neddylation promotes tumorigenesis, which can be targeted by neddylation inhibitors. (**B**) Other human diseases: Dysregulated neddylation is also involved in many other human diseases, as indicated.

The neddylation pathway is frequently hyperactivated in multiple cancer types, including lung (70), liver (71), colorectal (72), breast (73), esophageal squamous cell carcinoma (74), intrahepatic cholangiocarcinoma (75), and glioblastoma (76), often driven by overexpression of NEDD8 or neddylation-catalyzing enzymes. Clinically, neddylation hyperactivation is positively correlated with poor survival of patients with cancer, and elevated expression of neddylation enzymes serves as an independent predictor of reduced survival in several cancer cohorts (77). The consistent upregulation of neddylation components across diverse malignancies underscores their fundamental role in promoting tumorigenesis and positions them as attractive therapeutic targets as well as diagnostic biomarkers (3, 78, 79) (Figure 2A).

### Neddylation promotes tumorigenesis.

Hyperactivation of the neddylation pathway promotes tumorigenesis by producing sustained activation of CRLs, which in turn ubiquitylate and degrade critical tumor suppressor proteins, such as p21, p27 and p53, thus accelerating cell cycle progression and uncontrolled growth (70, 80–82). Beyond canonical K48-linked degradation, CRLs also catalyze noncanonical ubiquitin linkages, most notably K63 chains, which typically alter substrate trafficking or signaling without causing destruction (83). Neddylation-driven CRL activation directly influences ubiquitin linkage selection: in cancer, hyperneddylation enhances K48-linked ubiquitylation to degrade tumor suppressors (70, 80–82), while also promoting K63-linked ubiquitylation to stabilize oncoproteins such as Akt, thereby fostering oncogenic signaling and therapy resistance (84). This dual regulation positions neddylation as a central signaling hub for tumor initiation, progression, and metastasis beyond mere protein turnover. Neddylation further supports cancer cell survival by inhibiting apoptosis via targeting the proapoptotic protein NOXA for degradation and blocking the extrinsic apoptosis pathway (74, 85). Neddylation directly modifies and stabilizes several oncogenic proteins, including HER2 (73), Hu antigen R (HuR) (86), and the small GTPase RHEB (87). Furthermore, neddylation facilitates metabolic reprogramming (51), DNA damage response evasion (88), and therapeutic resistance across various cancers (19). Finally, by modulating SOX2 levels via CRL1^FBXW2^, neddylation regulates cancer stem cells, which are critical for tumor initiation, metastasis, and relapse (89, 90).

Deneddylating enzymes are also implicated in tumorigenesis. For example, elevated CSN subunit 5 (CSN5) promotes p27 degradation through SCF^Skp2^ hyperactivation, driving G_1_/S progression and proliferation. Furthermore, CSN5 stabilizes oncoproteins such as HIF-1α and c-Jun by facilitating CRL recycling, thereby enhancing angiogenesis and inflammatory signaling (91).

### Targeting neddylation for anticancer therapy: preclinical evidence.

Genetic evidence from tissue-specific knockout mouse studies supports neddylation as a promising therapeutic target. For instance, in a *Kras^G12D^*-driven lung cancer model, *Rbx2* deletion markedly suppressed tumorigenesis by activating multiple tumor suppressor pathways, including accumulation of p21, p27, NOXA, IκBα, and DEPTOR (92). In the *Kras^G12D^* pancreatic ductal adenocarcinoma model, *Ube2f* deletion blocked acinar-to-ductal metaplasia and PanIN progression by disrupting CRL5^Asb11^-mediated ubiquitylation and degradation of Diras2, an inhibitor of RAS, thereby inactivating the MAPK–c-Myc axis (93). Liver-specific *Ube2f* knockout alleviated steatosis and tumorigenesis induced by *Pten* loss through inactivation of the mTORC1 signal via blocking of Rheb neddylation (87), and prostate-specific *Rbx2* knockout suppresses prostate tumorigenesis induced by *Pten* loss via PI3K/AKT/mTOR inactivation (94).

The antitumor efficacy of small molecule–mediated neddylation inhibition has been firmly established across diverse preclinical models, including cell lines, xenografts, genetically engineered mouse models, and patient-derived xenografts. In models of lung cancer, the NAE inhibitor MLN4924 suppresses proliferation, migration, and motility of cancer cells in vitro and inhibits tumor formation and metastasis in xenograft models (70). MLN4924 alone or with the estrogen receptor degrader fulvestrant markedly inhibits ER-positive breast cancer growth both in vitro and in vivo (95). Combining MLN4924 with inhibitors of PKM2, ASCT2, or SLC7A11 effectively blocks breast cancer growth, highlighting a promising combinational targeting strategy (44, 51, 53). Combining MLN4924 with the TOP1 inhibitor irinotecan has synergistic effects in patient-derived organoid and xenograft models of colorectal cancer (CRC) (88). The NAE inhibitor compound 26 also exhibits potent antitumor activity in CRC and leukemia xenografts (96). MLN4924 exerts antitumor effects via CRL inactivation and induction of autophagy and apoptosis in a xenograft model of liver cancer (97). MLN4924 also suppresses tumor growth in subcutaneous esophageal cancer xenografts without notable toxicity (74) and suppresses orthotropic glioma xenograft growth (76). Importantly, MLN4924 upregulates PD-L1 expression in malignant cells, indicating potential synergistic benefits when combined with immune checkpoint blockade therapies (98, 99).

## Other human diseases

Beyond cancer, dysregulated neddylation is emerging as a key pathological promoter in a wide range of nonmalignant diseases in heart and liver as well as immune-related infectious, metabolic, and neurodegenerative diseases (Figure 2B).

### Cardiac development and chemotherapy-induced cardiac injury.

Neddylation is essential for heart development and homeostasis, with its components highly expressed in embryonic hearts but declining postnatally (27, 100). Disruption of this pathway, whether through perinatal interference or cardiomyocyte-specific *Nae1* deletion, impairs cardiac development and leads to lethal cardiomyopathy in mice (27, 101). In the adult mouse heart, genetic deletion of *Rbx2* represses mitophagy, facilitates the accumulation of damaged mitochondria, and perturbs metabolic homeostasis, ultimately triggering dilated cardiomyopathy and heart failure (102). In contrast to this requirement for normal development and function, neddylation may be hyperactivated in the setting of cardiotoxicity. MLN4924 has shown therapeutic potential in mitigating doxorubicin-induced cardiotoxicity in mice by preserving mitochondrial function, reducing apoptosis and oxidative stress, and improving contractile performance of cardiomyocytes, while limiting fibrosis (103). Thus, targeting neddylation appears to be a promising strategy for reducing doxorubicin-induced cardiac injury.

### Liver diseases.

The essential role of neddylation in liver biology is underscored by the finding that liver-specific knockout of *Nedd8* or *Uba3* in mice results in postnatal lethality accompanied by fatty liver and cellular senescence (45). However, while neddylation is indispensable for normal liver development, its chronic or excessive activation is increasingly recognized as a driver of liver pathology. Indeed, in liver disease states, neddylation is broadly upregulated. For example, both NAE1 expression and global neddylation are elevated during human liver fibrosis, whether it originates from hepatitis B infection or alcohol abuse (104). Mechanistically, neddylation of EphB1 enhances its kinase activity, promoting hepatic stellate cell activation in a mouse model of CCl4-induced fibrosis (105). Neddylation of mitochondrial electron transfer proteins (ETFA and ETFB) protects against fasting-induced steatosis, whereas its dysregulation promotes metabolic dysfunction–associated progression of steatotic liver disease (MASLD) (45). Consistent with this finding, MLN4924 enhances fatty acid oxidation and reduces steatosis in an MASLD mouse model, suggesting therapeutic potential (106). Neddylation also contributes to disease progression through posttranscriptional regulation: modification of SRSF3 at lysine 11 promotes its degradation, which is implicated in the progression of metabolic dysfunction–associated steatohepatitis and cirrhosis (107). Furthermore, neddylation is dramatically upregulated in acetaminophen-induced liver injury (AILI), a common cause of acute liver failure, and MLN4924 ameliorated hepatic necrosis and promoted liver regeneration in both clinical biopsies and mouse models of AILI, indicating its potential as a novel therapeutic agent for this disease (108). Similarly, DI-1859, an inhibitor of the co-E3 ligase DCN1, protects mice from AILI by causing NRF2 accumulation (109). Collectively, targeting overactivated neddylation could offer a novel therapeutic strategy across liver diseases.

### Immune-related infection and inflammatory diseases.

Neddylation exerts context-dependent effects on immunity, acting as a double-edged sword that requires precise regulation. In innate immunity, neddylation is essential for antiviral defense. For example, in mouse models, *Nedd8* or *Ube2m* deficiency compromises resistance to viruses including HSV-1 (110, 111). Conversely, neddylation overactivation is detrimental in the setting of bacterial infection, and its inhibition, whether through *Ube2m* deletion or MLN4924 treatment, curbs excessive inflammation in sepsis models and restores vascular function by preserving RhoA signaling (112, 113). This regulatory role extends to the chronic infection setting. In *Staphylococcus aureus* biofilm-associated periprosthetic joint infection (PJI), neddylation drives the expansion of immunosuppressive myeloid-derived suppressor cells and M2 macrophages (114); the neddylation inhibitor TAS4464 reverses these effects (114).

Neddylation also drives inflammation via CRL-mediated IκB degradation and NF-κB activation, thus promoting proinflammatory cytokine production (115). In a rat model of diabetic retinopathy, cullin-3 neddylation promotes Nrf2 degradation, exacerbating ROS-induced disruption of blood-retinal barrier, and MLN4924 treatment attenuates retinal damage and inflammation in diabetic rats, highlighting its therapeutic promise (116). In cerebral ischemia, Nae1, Uba3, and Ube2m are upregulated, and MLN4924 treatment reduces neutrophil infiltration and preserves integrity of the blood-brain barrier, thus mitigating brain injury (117). In the same ischemia model, Rbx2 is induced, and injection of a recombinant adenoviral vector expressing human RBX2 protects against brain damage induced by ischemia and reperfusion (118).

In the setting of autoimmune diseases, neddylation regulates pathogenic immune responses. For example, in a murine model of lupus, neddylation promotes double-negative (DN) T cell accumulation, whereas *Ube2m* deletion or MLN4924 treatment reduces DN T cells and attenuates the disease (119). NAE1 is upregulated in CD4^+^ T cells from patients with multiple sclerosis, and MLN4924 alleviates experimental autoimmune encephalomyelitis (120). We recently found that deletion of *Rbx1* or *Ube2m* in Tregs causes an early-onset fatal autoimmune disorder in mice, indicating their critical role in Treg fitness (43, 121).

### Metabolic diseases.

Clinical data show elevated expression of NAE1, NEDD8, and neddylated cullin proteins in individuals with obesity and T2DM, indicating overactivation of the neddylation pathway (46, 122). Mechanistically, neddylation promotes adipogenesis and lipid accumulation by stabilizing PPARγ (48). Our collaborative study further identified that UBE2M promotes TRIM21 neddylation, leading to VHL degradation and subsequent HIF-1α accumulation, driving IL-1β production in macrophages and exacerbating obesity-induced inflammation (50). MLN4924 treatment blocks PPARγ neddylation and reduces high-fat diet–induced obesity in mice (48).

### Neurodegenerative disorders.

Physiological neddylation supports neuronal function, and its disruption exacerbates neurodegeneration (123). A whole-genome CRISPR screen in a human stem cell model of Alzheimer’s disease (AD) identified neddylation as a modulator of cellular aging and neurodegeneration (124). In hippocampal neurons from patients with AD, NEDD8 mislocalizes to the cytoplasm. NAE1 is involved in amyloid precursor protein processing, and neddylation of presenilin promotes amyloid-β production, a hallmark of AD pathology (125–127). Furthermore, relevant to Parkinson’s disease (PD), neddylation regulates Parkin E3 ligase activity (128), and its dysregulation leads to accumulation of toxic proteins, which contributes to PD pathogenesis (129).

## Small-molecule inhibitors of neddylation enzymes

Given the essential role of neddylation in cellular homeostasis and its association, upon abnormal activation, with many human diseases, targeting neddylation presents an attractive therapeutic strategy. Since the discovery of the first neddylation inhibitor, MLN4924 (pevonedistat), in 2009 (18), the development of small-molecule inhibitors targeting neddylation enzymes has advanced rapidly, and these inhibitors have become the core of neddylation-targeted therapies (130). Neddylation therapeutics can be subcategorized into inhibitors of E1 (NAE), E2 (UBE2M or UBE2F, including E2-E3 interactions), or E3 (RBX1 or RBX2) and deneddylases (Table 1).

### E1 (NAE) inhibitors.

As the rate-limiting enzyme of neddylation cascade, NAE is the most well-characterized potential therapeutic target. The NAE inhibitor MLN4924 forms a covalent adduct with NEDD8 that mimics the NEDD8-AMP intermediate, thereby blocking NAE activity and the downstream neddylation cascade (131). This inhibition leads to accumulation of CRL tumor suppressive substrates and triggers anticancer effects (e.g., cell cycle arrest, apoptosis, senescence, autophagy) in a cell type–dependent manner (3). MLN4924 has demonstrated broad antitumor activity in preclinical models of both hematological and solid malignancies (132).

However, MLN4924 exhibits several neddylation-independent “off-target” effects that may broaden its therapeutic potential, while also posing some unanticipated challenges. Examples include inducing EGFR dimerization to activate RAS/MAPK and PI3K/AKT signaling, enhancing glycolysis by promoting PKM2 tetramerization, attenuating IL-17A–induced NF-κB activation and sensitizing cancer cells to TRAIL-induced apoptosis (for review, see ref. 133).

TAS4464, a structural analog of MLN4924, is a more selective and potent NAE inhibitor, with an IC_50_ value of 0.96 nM. TAS4464 exerts greater inhibitory effects than MLN4924 in both enzyme assays and cells, making it a promising agent for treating hematologic and solid tumors (134, 135).

Beyond MLN4924 and TAS4464, numerous structural analogs of MLN4924, such as compound 2, compound 7, ABP1, and ABP A3, have been identified (130). Other classes of compounds with moderate-to-high NAE-inhibitory activity include nonadenosine sulfamate analogs (e.g., LZ3 and HA-1141) and noncovalent inhibitors (e.g., the natural product 6,6’-biapigenin and rhodium [III] complexes), all of which are still in the preclinical stage (130).

HA-1141, a small molecule that directly binds to UBA3, was identified through virtual screening targeting the UBA3-UBE2F interface (136). HA-1141 exerts dual antitumor mechanisms by canonically inhibiting E1 to block neddylation of CUL1–CUL5, while unexpectedly inducing a noncanonical ER stress response that activates the PKR-ATF4-ISR axis to inhibit protein synthesis and mTORC1 activity. This combined effect potently inhibits proliferation of lung cancer cells and tumor growth in xenograft models (136).

### E2 inhibitors.

Targeting the E2-conjugating enzymes UBE2M and UBE2F provides a strategy for branch-specific inhibition of neddylation. While direct inhibition of these enzymes has been hampered by their structural dynamics and shallow active sites (137), recent advances have focused on disrupting E1-E2 (UBA3-UBE2F) and E2-E3 (UBE2M-DCN1) interactions, offering a promising alternative approach for inhibitor discovery (136, 138, 139). HA-9104, discovered via virtual screening of the UBA3-UBE2F interface, binds UBE2F to block CUL5 neddylation, leading to NOXA accumulation and apoptosis. It also forms DNA adducts via its 7-azaindole group, causing DNA damage and G_2_/M arrest, and sensitizes lung cancer cells to radiation (139).

DCN1 forms a complex with UBE2M to enhance cullin neddylation (140). The prototypical inhibitor NAcM-HIT and its optimized analog NAcM-OPT specifically disrupt this interaction and inhibit neddylation of CUL1 and CUL3 without affecting other cullins (138, 141). Subsequent development has yielded more potent and selective tools, including WS-383, a highly potent reversible inhibitor (IC_50_ = 11 nM) that stabilizes CRL substrates, including p21, p27, and NRF2 (142), and the DI-series compounds (DI-591, DI-404), which exhibit exquisite selectivity for DCN1 and inhibit CUL3 neddylation without cytotoxicity (143, 144). Follow-up studies led to the discovery of covalent inhibitors DI-1548 and DI-1859, which achieve low nanomolar potency (up to 1,000-fold greater than their predecessor); DI-1859 shows in vivo efficacy by protecting against AILI via NRF2 stabilization (109, 145). A recent review provides a complete list of DCN1 inhibitors (6). Collectively, these E2-E3 interaction inhibitors provide valuable tools for dissecting branch-specific neddylation functions and offer a more targeted therapeutic alternative to upstream E1 inhibitors.

### E3 inhibitors.

Targeting neddylation E3 ligases presents greater challenges than inhibiting E1 or E2 enzymes, primarily due to the difficulties of disrupting protein-protein interactions and the diversity of E3 family members (130). Through a pilot AlphaScreen-based high-throughput screen (HTS) for inhibitors of CUL5 neddylation, catalyzed by NAE/UBE2F/RBX2, we identified gossypol, a phenolic compound derived from the cotton plant, as a first-in-class inhibitor that binds both RBX1 and RBX2, blocks neddylation of CUL1 and CUL5, and stabilizes NOXA and MCL-1, leading to synergistic tumor suppression when combined with MCL-1 inhibitors (146). Clinical candidates remain elusive, but emerging chemical probes targeting specific CRLs offer proof of concept for this underexplored therapeutic avenue (147).

### Dual-targeting inhibitors.

Dual-targeting inhibitors that simultaneously block neddylation and other oncogenic pathways represent an emerging strategy to enhance efficacy and circumvent resistance. WS-384 was reported as an orally active dual inhibitor of DCN1/UBE2M and the demethylase LSD1, which effectively induces cell cycle arrest, DNA damage, and apoptosis in non–small cell lung cancer (NSCLC) models (148). However, future rational combinations of neddylation inhibitors with other anticancer drugs should be mechanism-driven, ideally via a synthetic lethality-like mechanism for maximal efficacy.

## Small-molecule inhibitors of deneddylation enzymes

### CSN5 inhibitors.

Several types of CSN5 inhibitors have been reported. High-throughput screening identified a weak inhibitor (CSN5i-1a), cocrystal structure-guided optimization of which yielded CSN5i-2, and further pharmacokinetics (PK) optimization produced CSN5i-3 (149). CSN5i-3 inhibits cullin-1 deneddylation with an IC_50_ of 5.8 nM, exhibits excellent selectivity and oral bioavailability, and suppresses growth of cancer cells and xenograft tumors (149). Notably, CSN5i-3 acts via an unprecedented “orthosteric molecular glue” mechanism: it binds cooperatively with NEDD8-conjugated substrate to the CSN active site, coordinating catalytic zinc and contacting both CSN5 and NEDD8, thus achieving high potency and selectivity without intrinsic affinity for the free enzyme (150). In addition to CSN5i-3, 2-aminothiazole-4-carboxylic acids were identified as CSN5 inhibitors that downregulate PD-L1 in cancer cells, whereas shikonins were found to be nanomolar CSN5 inhibitors that upregulate PD-L1 in cancer cells (151). Most recently, an azaindole metallo-CSN5 inhibitor was reported with nanomolar CSN5 inhibitory activity, which increased cullin-1 neddylation and showed synergistic anticancer effects with PARP inhibitors by enhancing DNA damage (152). Moreover, targeting deneddylation indirectly by modulating CRL-CSN complex formation with small molecules can regulate CRL neddylation and activation, thereby sensitizing cancer cells to the neddylation inhibitor MLN4924 (153).

### SENP8 inhibitors.

Through virtual HTS, five active SENP8 inhibitor candidates have been identified, with PubChem CIDs of 17300927, 2957665, 2955496, 17299262, and 1109711. These molecules are awaiting further validation and characterization (154).

### UCH-L1/UCH-L3 inhibitors.

TCID (4,5,6,7-Tetrachloroindan-1,3-dione), a selective UCH-L3 inhibitor, was identified from a UCH-L1–targeted screen. The compound has an IC_50_ = 0.6 μM against UCH-L3 with approximately 125-fold selectivity over UCH-L1 (155). Since both UCH-L1 and UCH-L3 function as dual-function deubiquitinases/deneddylases (156, 157), TCID may serve as a dual inhibitor for both processes with unknown biological activities.

## Clinical trials of neddylation E1 inhibitors

While numerous preclinical studies have been conducted to evaluate the above described neddylation inhibitors (130), only two E1 inhibitors, MLN4924 (also known as pevonedistat in clinical trials) and TAS4464, have been advanced to clinical trials to date, and none of them has yet received FDA approval, highlighting the early stage of clinical translation of neddylation inhibitors (Table 2).

MLN4924 has been evaluated across a wide range of hematologic and solid tumors in phase I–III trials. The first-in-human phase I study, performed in patients with acute myeloid leukemia (AML) and myelodysplastic syndromes (MDS), established its safety, tolerability, and modest single-agent activity (158). Subsequent investigations in relapsed/refractory multiple myeloma and lymphoma (159), metastatic melanoma (160), and advanced nonhematologic malignancies (161) confirmed its clinical feasibility and consistent PK profile across ethnic groups (162).

PK studies showed that MLN4924 is primarily metabolized by CYP3A4 and is a P-glycoprotein substrate. However, precise drug-drug interaction studies revealed no clinically meaningful effects when coadministered with moderate or strong CYP3A/P-gp inhibitors (163) or with the CYP3A inducer rifampin (164). Metabolic clearance is the major elimination route, with near-complete recovery of a radiolabeled dose within one week, providing clinical dosing strategies (165).

In pediatric patients with cancer, MLN4924 combined with the chemotherapeutics azacitidine and fludarabine showed no meaningful antileukemic activity in relapsed/refractory AML (166). In contrast, its combination with irinotecan and temozolomide yielded promising responses in pediatric solid tumors, warranting further investigation (167). In adult AML, MLN4924 combined with azacitidine demonstrated preclinical synergy and encouraging activity in a phase Ib trial (168). This led to a global phase II study in higher-risk MDS, chronic myelomonocytic leukemia (CMML), or low-blast AML, where the combination improved survival, with the most pronounced benefit in the MDS subgroup (169). Based on these results, MLN4924 received FDA Breakthrough Therapy designation for higher-risk MDS (170). However, the subsequent phase III PANTHER trial failed to meet its primary endpoint, showing no improvement over azacitidine alone in higher-risk MDS/CMML (171). Similarly, a phase II study in TP53-mutated AML found no cytogenetic responses with this combination (172).

Several studies report investigations of MLN4924 combination therapies. In relapsed/refractory AML/MDS, MLN4924 combined with the HDAC inhibitor belinostat showed modest but notable activity (173). In advanced solid tumors, combinations with docetaxel or carboplatin/paclitaxel were well tolerated and yielded durable responses in pretreated patients (174). The triplet regimen of azacitidine, venetoclax, and MLN4924 demonstrated encouraging preliminary efficacy in relapsed/refractory AML (175) and in MDS or CMML after failure with hypomethylating agents (176). Additional trials were conducted with moderate efficacy in relapsed NSCLC when combined with docetaxel (177) and in platinum-refractory advanced gastric cancer when combined with capecitabine and oxaliplatin (178). Despite these efforts, Takeda did not announce plans to continue MLN4924 development during its investor conferences or in financial reports between 2022 and 2025 (179). Furthermore, no new clinical trials have been initiated since 2021, suggesting that the program may have been discontinued.

TAS4464, another NAE inhibitor, showed potent antitumor activity in preclinical hematologic and solid tumor models without causing marked weight loss in mice (135). A first-in-human phase I study was subsequently conducted in patients with advanced solid tumors to evaluate its safety, PK, pharmacodynamics (PD), and preliminary efficacy (180). However, the TAS4464 showed dose-limiting liver toxicity, and even at the highest doses tested (up to 56 mg/m²), the drug failed to reach pharmacologically active levels predicted from preclinical studies. These results precluded adequate assessment of its antitumor efficacy, and the trial was terminated (180).

## Challenges in clinical translation of neddylation inhibitors

**Figure 3:**
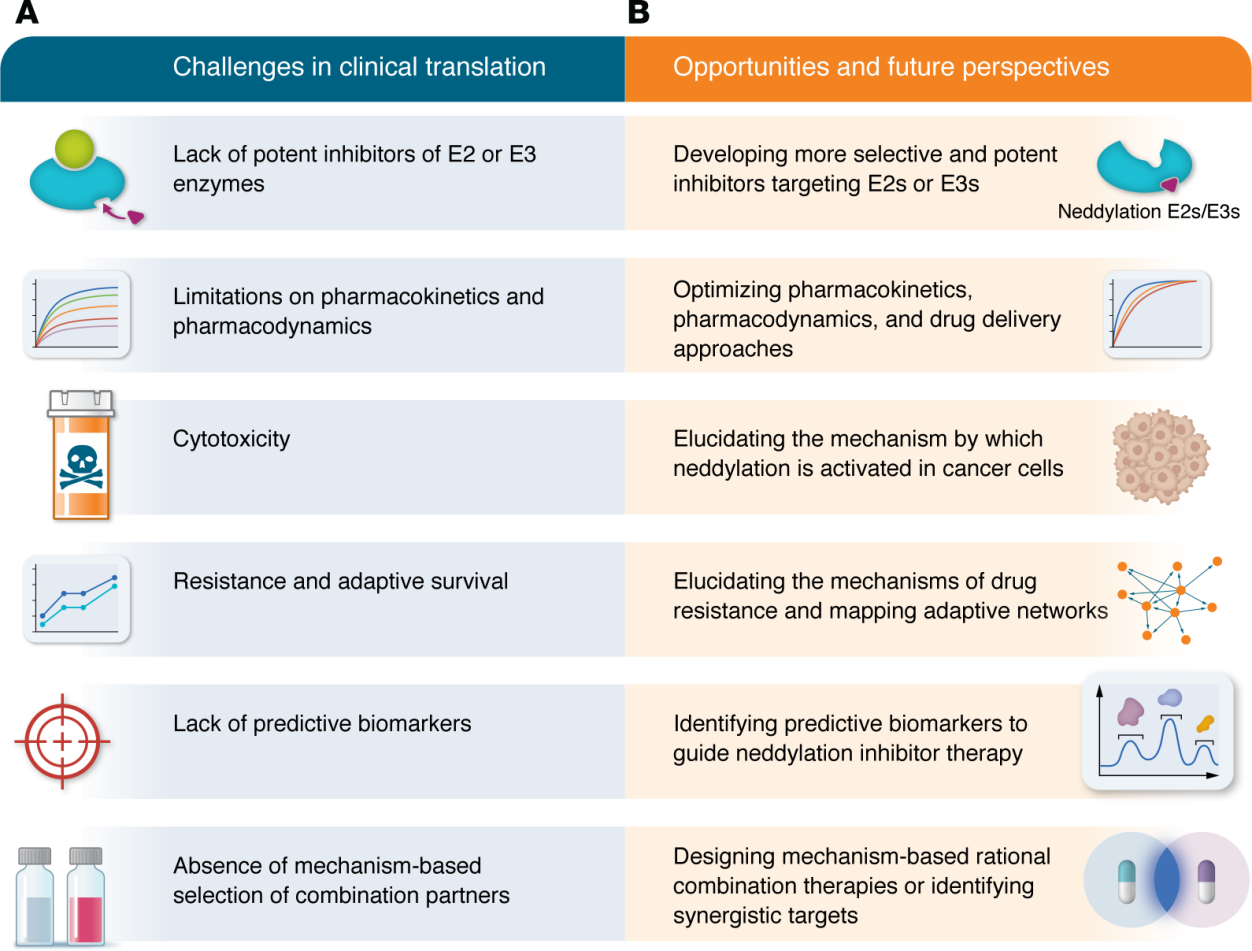
Challenges and opportunities for neddylation-targeted therapy. (**A**) Challenges and (**B**) opportunities for neddylation-targeted therapy.

Despite the strong promise of neddylation as an anticancer target, clinical translation has faced substantial hurdles, underscoring the need for deeper biological insight and strategic innovation (Figure 3A).

### Lack of potent and selective E2 or E3 inhibitors.

Currently, there are no potent and selective small-molecule inhibitors targeting the neddylation E2 enzyme UBE2F or the E3 ligase RBX2. While HA-9104 was reported as a UBE2F-based E2 inhibitor that blocks CUL5 neddylation, it has the off-target effect of triggering the DNA damage response by forming DNA adducts via its 7-azaindole group (139).

### Limitations on PK and PD.

Optimal properties of PK and PD are essential for clinical success, but neddylation inhibitors often fall short. On the PK side, early inhibitors like compound 7 (a DCN1-UBE2M inhibitor) demonstrated potent biochemical activity but suffered from rapid microsomal metabolism, leading to poor in vivo stability and low bioavailability (141). Although later analogs improved oral exposure and stability, issues with tissue penetration and target engagement remain. On the PD side, poor correlation between drug exposure and response complicates dose optimization (181). While neddylation inhibition increases CRL substrates (in theory for both tumor suppressors and oncogenic proteins), the selective accumulation of tumor suppressor proteins (such as p21, p27, NOXA) seen in preclinical studies does not reliably predict efficacy, and no standardized method exists to assess target engagement clinically. These uncertainties hinder optimal dosing and clinical translation.

### Therapeutic window.

As neddylation E1 inhibitors, both MLN4924 and TAS4464 block the entire neddylation pathway to inactivate all CRLs, leading to, in theory, the accumulation of hundreds of CRL substrates as well as many noncullin substrates. This universal blockage of neddylation modification could be the root of their cytotoxicity and highlights the need for selective inhibitors downstream of E1.

As noted above, dose-limiting hepatotoxicity emerged as a major obstacle for TAS4464, leading to its discontinuation in a phase I study (180). It is unclear why the toxicity occurs particularly in the liver. Advanced models, such as human liver organoids, will be essential to elucidate underlying mechanism of toxicity and to guide the design of safer next-generation inhibitors.

### Resistance and adaptive survival.

Development of drug resistance in tumor cells presents another key challenge. In breast cancer, *PTEN* loss confers resistance to MLN4924 (182). In addition, as expected, treatment-induced mutations in *UBA3* also confer resistance to MLN4924 (183). Other contributors to resistance and adaptive survival could be attributable to some tumor-promoting off-target effects of MLN4924, such as activation of the EGFR signal by triggering its dimerization (184) and enhancing glycolysis via PKM2 tetramerization to support survival of breast cancer cells (44). Mapping these context-dependent adaptive networks will be critical for developing effective combinatorial strategies.

### Lack of predictive biomarkers.

The absence of reliable predictive biomarkers represents a major barrier to precision oncology applications, limiting the identification of patients who would most likely benefit from neddylation inhibitors. Consequently, response rates remain low in unselected cohorts, limiting the translational application (158, 159, 162). Systematic proteomic and functional genomic approaches are urgently needed to identify and validate neddylation-related biomarkers for clinical decision-making.

### Mechanism-based selection of combination partners.

Given limited monotherapy efficacy, rational combination strategies represent a high-priority direction. Encouraging preclinical studies have shown that combining neddylation inhibitors with ABCG2 inhibitors can reverse resistance to MLN4924, while combinations with immunotherapies can enhance antitumor immune responses by modulating the tumor microenvironment (185). Mechanism-guided combinations with immunotherapies or other targeted agents, prioritized with refined preclinical models, will be critical to clinical success.

## Opportunities and future perspectives

Over the past decade, targeting protein neddylation has advanced from foundational biological discovery to early-stage clinical evaluation (3, 19). A growing body of evidence has established neddylation as a critical player in cancer and other human diseases, reinforcing its promise as a therapeutic target (3). The development of MLN4924, a first-in-class NAE inhibitor, provided an invaluable tool for dissecting neddylation biology and delivered clinical proof of concept in patients with cancer. Nevertheless, its modest single-agent activity and association with inherent cytotoxicity underscores the complexities of targeting such a fundamental pathway and highlights the urgent need for refined strategies.

Several opportunities or priorities emerge for advancing both mechanistic understanding and neddylation-targeted therapeutics in responses to the challenges (Figure 3B).

First, to improve efficacy and minimize off-target toxicity, the field must move beyond pan-E1 inhibition. Promising strategies include the development of selective inhibitors targeting downstream E2s, E3s, or protein-protein interactions such as DCN1-UBE2M (142). Furthermore, innovative modalities like proteolysis-targeting chimeras (PROTACs) and molecular glues could be leveraged to degrade core aberrantly activated components of the neddylation enzymes (e.g., NAE1, UBA3, UBE2M, UBE2F, RBX1, or RBX2) (186).

Second, potent inhibitors should be optimized via structure-activity relationship studies with better PK or PD, along with optimizing drug delivery approaches (e.g., prodrugs) (187).

Third, while it is well established that neddylation is overactivated in a variety of human cancers, there remains a dearth of knowledge regarding the precise underlying mechanisms at the levels of gene amplification, transcription, translation, or posttranslation. Elucidation of the mechanisms is required to guide cancer selective targeting.

Fourth, systematic efforts utilizing single-cell multi-omics and functional genomics are urgently needed to elucidate the underlying mechanisms of developing drug resistance and mapping the context-dependent adaptive networks.

Fifth, systematic efforts using global proteomics and functional genomics are needed to identify and validate predictive biomarkers in an effort to stratify patients who are most likely to benefit from neddylation inhibition.

Sixth, the mechanism-based rational combination therapies should be prioritized and optimized using advanced human-relevant models, such as organoids and organ-on-a-chip platforms, to better assess efficacy and toxicity prior to clinical translation. Identification and validation of synergistic targets via HTS of chemical libraries or genome-wide CRISPR screens should also be pursued.

Finally, broadening investigation to nononcological indications, including fibrotic diseases, viral infections, and aging-related disorders, will likely reveal novel therapeutic applications. The causal relationships must be established, given that current evidence linking neddylation dysregulation to these diseases remains largely correlative (104, 122). Genetically engineered mouse models with tissue-specific deletion or overexpression of key neddylation components should be developed to address this gap. Furthermore, despite accumulating preclinical evidence supporting the efficacy of MLN4924 in obesity, MASLD, lupus, and doxorubicin-induced cardiotoxicity (48, 103, 106, 119), no corresponding clinical trials have been initiated, underscoring an urgent need to advance these indications.

In conclusion, neddylation remains a target of substantial promise, but translating this approach into clinical therapies will require deeper biological insight, better drug design, and more sophisticated clinical strategies. The journey from fundamental discovery to clinical application continues, a testament to the enduring value of investing in basic and translational science.

## Conflict of interest

The authors have declared that no conflict of interest exists.

## Funding support

This work was funded in part by the National Natural Science Foundation of China.

- Grants U22A20317 and 92253203 to YS.
- Grants 82473005 and 82202838 to SZ.
- Grant 82102947 to HL.

## Supplementary Material

Supplemental data

## Figures and Tables

**Table 1:**
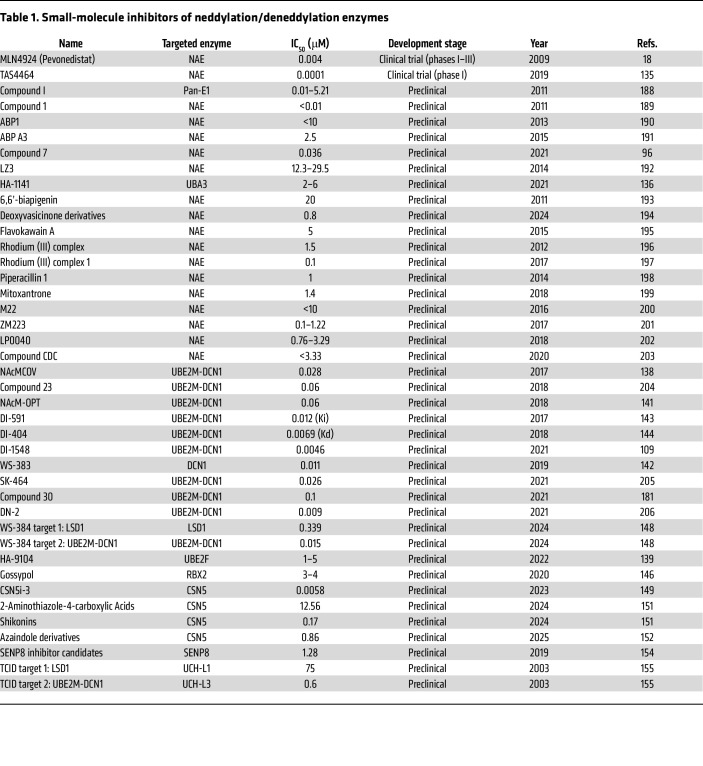
Small-molecule inhibitors of neddylation/deneddylation enzymes

**Table 2:**
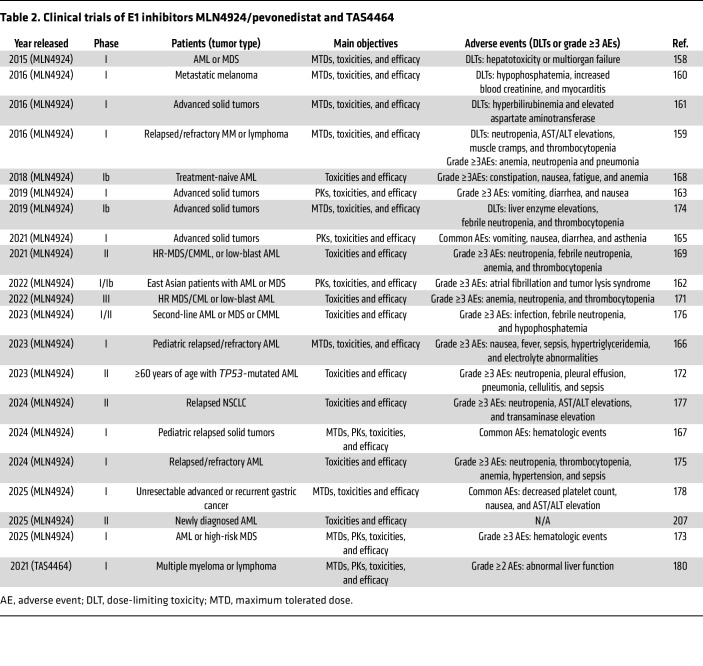
Clinical trials of E1 inhibitors MLN4924/pevonedistat and TAS4464

## Version 1 08/03/2026

Electronic publication
