# Supplementary material for: Protein neddylation as a therapeutic target: challenges and opportunities
Source: J Clin Invest. 2026 Aug 3;136(15):e206924. doi: 10.1172/JCI206924 (PMC13430020; doi:10.1172/JCI206924)
Supplement: Supplemental data [file jci-136-206924-s130.pdf]

Online supplementary material regarding the review manuscript

Protein neddylation as a therapeutic target: challenges and opportunities

Shizhen Zhang[1,2*], Huiyin Lan[3*], Yi Sun[1,4-7#]

[1]Cancer Institute and [2]Department of Breast Surgery of the Second Affiliated Hospital, Zhejiang University School of Medicine, Hangzhou 310029, China.

[3]Department of Radiation Oncology, Zhejiang Cancer Hospital; Hangzhou Institute of Medicine (HIM), Chinese Academy of Sciences; Zhejiang Key Laboratory of Particle Radiotherapy Equipment, Hangzhou 310022, China.

[4]Institute of Translational Medicine, Zhejiang University School of Medicine, Hangzhou 310029, China

[5]Zhejiang University Cancer Center, Hangzhou 310029, China

[6]Research Center for Life Science and Human Health, Binjiang Institute of Zhejiang University, Hangzhou 310053, China

[7]State Key Laboratory of Transvascular Implantation Devices, the Second Affiliated Hospital, Zhejiang University School of Medicine, Hangzhou 310029, China

* SZ and HL contributed equally to this work.

[#]Correspondence to: YS, yisun@zju.edu.cn

Table S1. Neddylation enzymes and NEDD8 substrates [1]

Neddylation enzymes	Substrates and references
**NEDD8-activating E1 enzymes**	
(NAE1/UBA3)	Akt (1), BCA3 (2), BRAP2 (3), CXCR5 (4), Cofilin 1 (5), DIC1 (6), DDX5 (7), EphB1 (8), ETFA/B (9), FoxO3a (10), Gadd45a (11), HDAC1 (12), HDAC2 (13), HDAC6 (14), HER2 (15), HIF1α (16), HIF2α (16), hnRNPA2B1 (17), LKB1 (1), mHTT (18), NRF2 (19), PCK1 (20), Pol η (21), RNF187 (22), RhoA (23), SRSF3 (24), TAK1 (11), TRAF6 (25)<br>Essentially all known and yet-to-be identified neddylation substrates
**NEDD8-conjugating enzymes E2**	
UBE2M	AICD (26), ARIH2 (27), BCR-ABL (28), Cullins 1-4 (29), Caspase-1 (30), cGAS (31), E2F1 (32, 33), H2A (34), HBx (35), Histone H4 (36), HSP70 (37), IRF3 (38), IRF7 (38), LSD1 (39), LC3B (40), MKK7 (41), MYD88 (42), NEDD4-2 (43, 44), NIK (45), NLRP3 (46), Parkin (47, 48), PARP1 (49), PCNA (50), PINK1 (47), PML (51), PSD-95 (52), PTEN (53), RCAN1 (54), RNF168 (34), Shc (55), SHP2 (56), SMURF1 (57), SMURF2 (58), TGFBR2 (59), p53 (60-62), TRF1 (63), TRIM21 (64), TRIM25 (65), USP39 (66), VHL (67, 68)
UBE2F	Coro1a (69), Cullin 5 (29), Cullin 9 (70), RHEB (71)
**Neddylation E3 ligases**	
c-CBL	c-Src (72), EGFR (73, 74), TGFBR2 (59), Parkin (47, 48), PARP1 (75)
cIAP1	p21 (76)
DCN1-5	Cullin1-5 (77)
FBX4(SCF)	TRF1 (63)
FBXO11(SCF)	p53 (62)
β-TrCP2(SCF)	β-catenin (78, 79)
Hakai	Ajuba (80)
HDM2/MDM2	HBx (35), PB2 (81), SREBP1C (82), HuR (83), MDM2 (60),<br>PPARγ (84), PSD-95 (52), RPL11 (85-87), RPS14 (88), RPS27/RPS27L (89), p53 (60), p73 (90), VHL (67, 68)
HUWE1	RPL7 (91)
Itch	JunB (92)
RAD18	PCNA (50), RAD18 (93)
RAPSYN	BCR-ABL (28)
RanBP2	MKK7 (41)
RBX1	Cullins 1-4 (29), Cullin 9 (70), LC3B (40)
RBX2	Cullin 5 (29), RHEB (71)
RNF111	cGAS (31), Histone H4 (36)
RNF168	H2A (34), RNF168 (34)
SMURF1	PDK1 (94), RRP9 (95), SMURF1 (57)
SMURF2	NLRP3 (46)
TRIM4	$3D^{pol}$ (96), CORO1A (97),
TRIM40	Coro1a (69), IKKγ (98)
Tfb3	yeast Cul4-type cullin Rtt101 (99)
XIAP	PTEN (53), SHP2 (56), VP2 (100)

[1]Note that proteins categorized as substrates of E1 and E2 enzymes are noted as such because the relevant E2 and/or E3 was unknown at the time of discovery. By definition, all NEDD8 substrates should be classified as substrates of E3 ligases.

Table S2. Deneddylases and their substrates

Deneddylases	Substrates and references
CSN	Cullins (101), EGFR (73, 74), HIF1α (16)
SENP8	Akt (1), BCA3 (AKIP1) (2), cGAS (31), Cofilin 1(CFL1) (5), Cullin 1-4 (102), c-Src (72), E2F1 (32, 33), Gadd45a (11), H2A (34), HBx (35), HDAC1 (12), HDAC2 (13), HSP70 (HSPA) (37), HuR (ELAVL1) (83), IRS1/2 (103), MDM2 (60, 104), MYD88 (42), NEDD4-2 (43, 44), NIK (MAP3K14) (45), Parkin (PRKN) (47, 48), PARP1 (49), PCNA (50), PINK1 (47), PML/RARα (51), PPARγ (PPARG) (84), PSD-95 (DLG4) (52), PTEN (53), RAD18 (93), RNF168 (34), RPL11 (85-87), RPS14 (88), RPS27/RPS27L (89), RRP9 (95), SHP2 (PTPN11) (56), SMURF1 (57), SMURF2 (58), SREBP1C (82), TAK1 (MAP3K7) (11), p53 (60), p73 (90), β-catenin (CTNNB1) (78, 79)
Ataxin-3	Unknown (105)
USP21	Unknown (106)
UCH-L1	Unknown (107)
UCH-L3	Unknown (108)
PfUCH54	Unknown (109)

References

1. Barbier-Torres L, Delgado TC, Garcia-Rodriguez JL, Zubiete-Franco I, Fernandez-Ramos D, Buque X, et al. Stabilization of LKB1 and Akt by neddylation regulates energy metabolism in liver cancer. *Oncotarget.* 2015;6(4):2509-23.
2. Gao F, Cheng J, Shi T, and Yeh ET. Neddylation of a breast cancer-associated protein recruits a class III histone deacetylase that represses NFkappaB-dependent transcription. *Nat Cell Biol.* 2006;8(10):1171-7.
3. Takashima O, Tsuruta F, Kigoshi Y, Nakamura S, Kim J, Katoh MC, et al. Brap2 regulates temporal control of NF-κB localization mediated by inflammatory response. *PLoS One.* 2013;8(3):e58911.
4. Renaudin X, Guervilly JH, Aoufouchi S, and Rosselli F. Proteomic analysis reveals a FANCA-modulated neddylation pathway involved in CXCR5 membrane targeting and cell mobility. *J Cell Sci.* 2014;127(Pt 16):3546-54.
5. Vogl AM, Phu L, Becerra R, Giusti SA, Verschueren E, Hinkle TB, et al. Global site-specific neddylation profiling reveals that NEDDylated cofilin regulates actin dynamics. *Nat Struct Mol Biol.* 2020;27(2):210-20.
6. Gupta D, and Maddika S. COP9 signalosome and PRMT5 methylosome complexes are essential regulators of Lis1-dynein-based transport. *Cell Rep.* 2026;45(1):116736.
7. Liang Y, Huang G, Zhu X, Huang C, Liu H, and Shu L. SIRT1 reduced DDX5 neddylation to attenuate myocardial ischemia/reperfusion injury. *Hum Cell.* 2026;39(2):43.
8. Li R, Zhang D, Han Y, Chen K, Guo W, Chen Y, et al. Neddylation of EphB1 Regulates Its Activity and Associates with Liver Fibrosis. *Int J Mol Sci.* 2023;24(4).
9. Zhang X, Zhang YL, Qiu G, Pian L, Guo L, Cao H, et al. Hepatic neddylation targets and stabilizes electron transfer flavoproteins to facilitate fatty acid β-oxidation. *Proceedings of the National Academy of Sciences of the United States of America.* 2020;117(5):2473-83.
10. Jankowski K, Lemay SE, Lozano-Ojalvo D, Pérez-Rodríguez L, Sauvaget M, Breuils-Bonnet S, et al. Pharmacological inhibition of Epac1 protects against pulmonary fibrosis by blocking FoxO3a neddylation. *Eur Respir J.* 2025;66(4).
11. Li S, Fang W, Cui Y, Shi H, Chen J, Li L, et al. Neddylation promotes protein translocation between the cytoplasm and nucleus. *Biochem Biophys Res Commun.* 2020;529(4):991-7.
12. Lai QY, He YZ, Peng XW, Zhou X, Liang D, and Wang L. Histone deacetylase 1 induced by neddylation inhibition contributes to drug resistance in acute myelogenous leukemia. *Cell Commun Signal.* 2019;17(1):86.
13. Pandey D, Hori D, Kim JH, Bergman Y, Berkowitz DE, and Romer LH. NEDDylation promotes endothelial dysfunction: a role for HDAC2. *J Mol Cell Cardiol.* 2015;81:18-22.
14. Nomura Y, Nakano M, Woo Sung H, Han M, and Pandey D. Inhibition of HDAC6 Activity Protects Against Endothelial Dysfunction and Atherogenesis in vivo: A Role for HDAC6 Neddylation. *Front Physiol.* 2021;12:675724.
15. Xia X, Hu T, He X, Liu Y, Yu C, Kong W, et al. Neddylation of HER2 Inhibits its Protein Degradation and promotes Breast Cancer Progression. *Int J Biol Sci.* 2023;19(2):377-92.
16. Ryu JH, Li SH, Park HS, Park JW, Lee B, and Chun YS. Hypoxia-inducible factor α subunit stabilization by NEDD8 conjugation is reactive oxygen species-dependent. *J Biol Chem.* 2011;286(9):6963-70.
17. Chen TR, Zhou JX, Hou YR, Zhang YN, Li CX, Zhu J, et al. Neddylation-Mediated hnRNPA2B1 Degradation Aggravates Retinal Endothelial Cell Dysfunction in Diabetic Retinopathy by Regulating miR-93-5p/VEGFA. *Invest Ophthalmol Vis Sci.* 2026;67(3):5.
18. Ghosh DK, and Ranjan A. HYPK coordinates degradation of polyneddylated proteins by autophagy. *Autophagy.* 2022;18(8):1763-84.

19. Rao W, Huang Y, Li J, Chen Q, Sun J, and Huang L. NEDD8 promotes the ferritinophagy and ferroptosis of neurons in ischemic stroke via mediating neddylation of NRF2. *J Stroke Cerebrovasc Dis.* 2026:108582.
20. Gonzalez-Rellan MJ, Fernández U, Parracho T, Novoa E, Fondevila MF, da Silva Lima N, et al. Neddylation of phosphoenolpyruvate carboxykinase 1 controls glucose metabolism. *Cell Metab.* 2023;35(9):1630-45.e5.
21. Moreno NC, Korchak EJ, Latancia MT, D'Orlando DA, Adegbenro T, Barnes RP, et al. Human DNA polymerase η is regulated by mutually exclusive mono-ubiquitination and mono-NEDDylation. *Nucleic Acids Res.* 2026;54(4).
22. Yang C, Liu X, Yang W, Wang L, Wang Z, Li J, et al. RNF187 neddylation in pancreatic cancer activates malignancy via IQGAP1-dependent actin cytoskeleton rearrangement. *Oncogene.* 2026;45(1):26-41.
23. Li XQ, Jin B, Liu SX, Zhu Y, Li N, Zhang QY, et al. Neddylation of RhoA impairs its protein degradation and promotes renal interstitial fibrosis progression in diabetic nephropathy. *Acta Pharmacol Sin.* 2025;46(6):1692-705.
24. Kumar D, Das M, Sauceda C, Ellies LG, Kuo K, Parwal P, et al. Degradation of splicing factor SRSF3 contributes to progressive liver disease. *J Clin Invest.* 2019;129(10):4477-91.
25. Liu K, Chen K, Zhang Q, Zhang L, Yan Y, Guo C, et al. TRAF6 neddylation drives inflammatory arthritis by increasing NF-κB activation. *Lab Invest.* 2019;99(4):528-38.
26. Lee MR, Lee D, Shin SK, Kim YH, and Choi CY. Inhibition of APP intracellular domain (AICD) transcriptional activity via covalent conjugation with Nedd8. *Biochem Biophys Res Commun.* 2008;366(4):976-81.
27. Gupta D, and Maddika S. A LisH-domain protein interaction map reveals a Lis1-ARIH2-dynein regulatory axis. *iScience.* 2025;28(11):113912.
28. Zhao M, Dai B, Li X, Zhang Y, Qiao C, Qin Y, et al. RAPSYN-mediated neddylation of BCR-ABL alternatively determines the fate of Philadelphia chromosome-positive leukemia. *Elife.* 2024;12.
29. Enchev RI, Schulman BA, and Peter M. Protein neddylation: beyond cullin-RING ligases. *Nat Rev Mol Cell Biol.* 2015;16(1):30-44.
30. Segovia JA, Tsai SY, Chang TH, Shil NK, Weintraub ST, Short JD, et al. Nedd8 regulates inflammasome-dependent caspase-1 activation. *Mol Cell Biol.* 2015;35(3):582-97.
31. Li C, Zhang L, Qian D, Cheng M, Hu H, Hong Z, et al. RNF111-facilitated neddylation potentiates cGAS-mediated antiviral innate immune response. *PLoS Pathog.* 2021;17(3):e1009401.
32. Loftus SJ, Liu G, Carr SM, Munro S, and La Thangue NB. NEDDylation regulates E2F-1-dependent transcription. *EMBO Rep.* 2012;13(9):811-8.
33. Aoki I, Higuchi M, and Gotoh Y. NEDDylation controls the target specificity of E2F1 and apoptosis induction. *Oncogene.* 2013;32(34):3954-64.
34. Li T, Guan J, Huang Z, Hu X, and Zheng X. RNF168-mediated H2A neddylation antagonizes ubiquitylation of H2A and regulates DNA damage repair. *J Cell Sci.* 2014;127(Pt 10):2238-48.
35. Liu N, Zhang J, Yang X, Jiao T, Zhao X, Li W, et al. HDM2 Promotes NEDDylation of Hepatitis B Virus HBx To Enhance Its Stability and Function. *J Virol.* 2017;91(16).
36. Ma T, Chen Y, Zhang F, Yang CY, Wang S, and Yu X. RNF111-dependent neddylation activates DNA damage-induced ubiquitination. *Mol Cell.* 2013;49(5):897-907.
37. Bailly AP, Perrin A, Serrano-Macia M, Maghames C, Leidecker O, Trauchessec H, et al. The Balance between Mono- and NEDD8-Chains Controlled by NEDP1 upon DNA Damage Is a Regulatory Module of the HSP70 ATPase Activity. *Cell reports.* 2019;29(1):212-24.e8.
38. Zhao M, Zhang Y, Yang X, Jin J, Shen Z, Feng X, et al. Myeloid neddylation targets IRF7 and promotes host innate immunity against RNA viruses. *PLoS Pathog.* 2021;17(9):e1009901.
39. Guo YJ, Pang JR, Zhang Y, Li ZR, Zi XL, Liu HM, et al. Neddylation-dependent LSD1

destabilization inhibits the stemness and chemoresistance of gastric cancer. *Int J Biol Macromol.* 2023;254(Pt 3):126801.
40. Xu L, Lyu X, Wang Y, Ni L, Li P, Zeng P, et al. Neddylation modification stabilizes LC3B by antagonizing its ubiquitin-mediated degradation and promoting autophagy in skin. *Proc Natl Acad Sci U S A.* 2025;122(15):e2411429122.
41. Zhu T, Wang J, Pei Y, Wang Q, Wu Y, Qiu G, et al. Neddylation controls basal MKK7 kinase activity in breast cancer cells. *Oncogene.* 2016;35(20):2624-33.
42. Yan F, Guan J, Peng Y, and Zheng X. MyD88 NEDDylation negatively regulates MyD88-dependent NF-κB signaling through antagonizing its ubiquitination. *Biochem Biophys Res Commun.* 2017;482(4):632-7.
43. Zhao J, Zhang B, Lai G, Xu R, Chu G, and Zhao Y. 20-Hydroxyeicosatetraenoic acid regulates the expression of Nedd4-2 in kidney and liver through a neddylation modification pathway. *Mol Med Rep.* 2017;16(6):9671-7.
44. Tu J, Zhang B, Fang G, Chang W, and Zhao Y. Neddylation-mediated Nedd4-2 activation regulates ubiquitination modification of renal NBCe1. *Exp Cell Res.* 2020;390(2):111958.
45. Xu C, Zhou H, Jin Y, Sahay K, Robicsek A, Liu Y, et al. Hepatic neddylation deficiency triggers fatal liver injury via inducing NF-κB-inducing kinase in mice. *Nat Commun.* 2022;13(1):7782.
46. Gai W, Wu M, Wu A, Jing Z, Ye Z, Jin J, et al. Neddylation Targets and Stabilizes NLRP3 to Augment Inflammasome-Mediated Colitis and Mood Disorder. *Adv Sci (Weinh).* 2026:e05906.
47. Choo YS, Vogler G, Wang D, Kalvakuri S, Iliuk A, Tao WA, et al. Regulation of parkin and PINK1 by neddylation. *Hum Mol Genet.* 2012;21(11):2514-23.
48. Um JW, Han KA, Im E, Oh Y, Lee K, and Chung KC. Neddylation positively regulates the ubiquitin E3 ligase activity of parkin. *J Neurosci Res.* 2012;90(5):1030-42.
49. Keuss MJ, Hjerpe R, Hsia O, Gourlay R, Burchmore R, Trost M, et al. Unanchored tri-NEDD8 inhibits PARP-1 to protect from oxidative stress-induced cell death. *EMBO J.* 2019;38(6).
50. Guan J, Yu S, and Zheng X. NEDDylation antagonizes ubiquitination of proliferating cell nuclear antigen and regulates the recruitment of polymerase η in response to oxidative DNA damage. *Protein & cell.* 2018;9(4):365-79.
51. Shao X, Chen Y, Xu A, Xiang D, Wang W, Du W, et al. Deneddylation of PML/RARα reconstructs functional PML nuclear bodies via orchestrating phase separation to eradicate APL. *Cell Death Differ.* 2022;29(8):1654-68.
52. Vogl AM, Brockmann MM, Giusti SA, Maccarrone G, Vercelli CA, Bauder CA, et al. Neddylation inhibition impairs spine development, destabilizes synapses and deteriorates cognition. *Nat Neurosci.* 2015;18(2):239-51.
53. Xie P, Peng Z, Chen Y, Li H, Du M, Tan Y, et al. Neddylation of PTEN regulates its nuclear import and promotes tumor development. *Cell Res.* 2021;31(3):291-311.
54. Noh EH, Hwang HS, Hwang HS, Min B, Im E, and Chung KC. Covalent NEDD8 conjugation increases RCAN1 protein stability and potentiates its inhibitory action on calcineurin. *PLoS One.* 2012;7(10):e48315.
55. Jin HS, Liao L, Park Y, and Liu YC. Neddylation pathway regulates T-cell function by targeting an adaptor protein Shc and a protein kinase Erk signaling. *Proc Natl Acad Sci U S A.* 2013;110(2):624-9.
56. Li Y, Zhou H, Liu P, Lv D, Shi Y, Tang B, et al. SHP2 deneddylation mediates tumor immunosuppression in colon cancer via the CD47/SIRPα axis. *J Clin Invest.* 2023;133(4).
57. Xie P, Zhang M, He S, Lu K, Chen Y, Xing G, et al. The covalent modifier Nedd8 is critical for the activation of Smurf1 ubiquitin ligase in tumorigenesis. *Nat Commun.* 2014;5:3733.
58. Shu J, Liu C, Wei R, Xie P, He S, and Zhang L. Nedd8 targets ubiquitin ligase Smurf2 for neddylation and promote its degradation. *Biochem Biophys Res Commun.* 2016;474(1):51-6.

59. Zuo W, Huang F, Chiang YJ, Li M, Du J, Ding Y, et al. c-Cbl-mediated neddylation antagonizes ubiquitination and degradation of the TGF-β type II receptor. *Mol Cell.* 2013;49(3):499-510.
60. Xirodimas DP, Saville MK, Bourdon JC, Hay RT, and Lane DP. Mdm2-mediated NEDD8 conjugation of p53 inhibits its transcriptional activity. *Cell.* 2004;118(1):83-97.
61. Singh RK, Iyappan S, and Scheffner M. Hetero-oligomerization with MdmX rescues the ubiquitin/Nedd8 ligase activity of RING finger mutants of Mdm2. *J Biol Chem.* 2007;282(15):10901-7.
62. Abida WM, Nikolaev A, Zhao W, Zhang W, and Gu W. FBXO11 promotes the Neddylation of p53 and inhibits its transcriptional activity. *J Biol Chem.* 2007;282(3):1797-804.
63. Jeong YY, Her J, and Chung IK. NEDD8 ultimate buster-1 regulates the abundance of TRF1 at telomeres by promoting its proteasomal degradation. *FEBS Lett.* 2016;590(12):1776-90.
64. Lu X, Kong X, Wu H, Hao J, Li S, Gu Z, et al. UBE2M-mediated neddylation of TRIM21 regulates obesity-induced inflammation and metabolic disorders. *Cell Metab.* 2023;35(8):1390-405 e8.
65. Zheng B, Qian F, Wang X, Wang Y, Zhou B, and Fang L. Neddylation activated TRIM25 desensitizes triple-negative breast cancer to paclitaxel via TFEB-mediated autophagy. *J Exp Clin Cancer Res.* 2024;43(1):177.
66. Wang Z, Wang Y, Chen Y, Shen H, Lu Y, Tong Y, et al. UBE2M as a bridge spanning neddylation and cell cycle regulation in colorectal adenocarcinoma. *Exp Mol Med.* 2026.
67. Russell RC, and Ohh M. NEDD8 acts as a 'molecular switch' defining the functional selectivity of VHL. *EMBO Rep.* 2008;9(5):486-91.
68. Wolf ER, Mabry AR, Damania B, and Mayo LD. Mdm2-mediated neddylation of pVHL blocks the induction of antiangiogenic factors. *Oncogene.* 2020;39(29):5228-39.
69. Fei X, Li Z, Yang D, Kong X, Lu X, Shen Y, et al. Neddylation of Coro1a determines the fate of multivesicular bodies and biogenesis of extracellular vesicles. *J Extracell Vesicles.* 2021;10(12):e12153.
70. Horn-Ghetko D, Hopf LVM, Tripathi-Giesgen I, Du J, Kostrhon S, Vu DT, et al. Noncanonical assembly, neddylation and chimeric cullin-RING/RBR ubiquitylation by the 1.8 MDa CUL9 E3 ligase complex. *Nat Struct Mol Biol.* 2024;31(7):1083-94.
71. Zhang F, Xiong X, Li Z, Wang H, Wang W, Zhao Y, et al. RHEB neddylation by the UBE2F-SAG axis enhances mTORC1 activity and aggravates liver tumorigenesis. *Embo j.* 2025;44(4):1185-219.
72. Lee GW, Park JB, Park SY, Seo J, Shin SH, Park JW, et al. The E3 ligase C-CBL inhibits cancer cell migration by neddylating the proto-oncogene c-Src. *Oncogene.* 2018;37(41):5552-68.
73. Oved S, Mosesson Y, Zwang Y, Santonico E, Shtiegman K, Marmor MD, et al. Conjugation to Nedd8 instigates ubiquitylation and down-regulation of activated receptor tyrosine kinases. *J Biol Chem.* 2006;281(31):21640-51.
74. Najor NA, Fitz GN, Koetsier JL, Godsel LM, Albrecht LV, Harmon R, et al. Epidermal Growth Factor Receptor neddylation is regulated by a desmosomal-COP9 (Constitutive Photomorphogenesis 9) signalosome complex. *Elife.* 2017;6.
75. Kwon DH, Shin S, Nam YS, Choe N, Lim Y, Jeong A, et al. CBL-b E3 ligase-mediated neddylation and activation of PARP-1 induce vascular calcification. *Exp Mol Med.* 2024;56(10):2246-59.
76. Chen SM, Lin TK, Tseng YY, Tu CH, Lui TN, Huang SF, et al. Targeting inhibitors of apoptosis proteins suppresses medulloblastoma cell proliferation via G2/M phase arrest and attenuated neddylation of p21. *Cancer Med.* 2018;7(8):3988-4003.
77. Zhou W, Xu C, Zhang S, Chen X, Zhou T, Liu T, et al. DCN-type NEDD8 E3 ligases: Structure, biological function and small-molecule inhibitor. *Pharmacol Res.* 2026;225:108151.
78. Wang B, Wang T, Zhu H, Yan R, Li X, Zhang C, et al. Neddylation is essential for β-

catenin degradation in Wnt signaling pathway. *Cell Rep.* 2022;38(12):110538.
79. Zhang L, Jing H, Li H, Chen W, Luo B, Zhang H, et al. Neddylation is critical to cortical development by regulating Wnt/β-catenin signaling. *Proc Natl Acad Sci U S A.* 2020;117(42):26448-59.
80. Liu M, Jiang K, Lin G, Liu P, Yan Y, Ye T, et al. Ajuba inhibits hepatocellular carcinoma cell growth via targeting of β-catenin and YAP signaling and is regulated by E3 ligase Hakai through neddylation. *J Exp Clin Cancer Res.* 2018;37(1):165.
81. Zhang T, Ye Z, Yang X, Qin Y, Hu Y, Tong X, et al. NEDDylation of PB2 Reduces Its Stability and Blocks the Replication of Influenza A Virus. *Scientific reports.* 2017;7:43691.
82. Ju UI, Jeong DW, Seo J, Park JB, Park JW, Suh KS, et al. Neddylation of sterol regulatory element-binding protein 1c is a potential therapeutic target for nonalcoholic fatty liver treatment. *Cell death & disease.* 2020;11(4):283.
83. Embade N, Fernandez-Ramos D, Varela-Rey M, Beraza N, Sini M, Gutierrez de Juan V, et al. Murine double minute 2 regulates Hu antigen R stability in human liver and colon cancer through NEDDylation. *Hepatology.* 2012;55(4):1237-48.
84. Park HS, Ju UI, Park JW, Song JY, Shin DH, Lee KH, et al. PPARγ neddylation essential for adipogenesis is a potential target for treating obesity. *Cell Death Differ.* 2016;23(8):1296-311.
85. Xirodimas DP, Sundqvist A, Nakamura A, Shen L, Botting C, and Hay RT. Ribosomal proteins are targets for the NEDD8 pathway. *EMBO Rep.* 2008;9(3):280-6.
86. Mahata B, Sundqvist A, and Xirodimas DP. Recruitment of RPL11 at promoter sites of p53-regulated genes upon nucleolar stress through NEDD8 and in an Mdm2-dependent manner. *Oncogene.* 2012;31(25):3060-71.
87. Sundqvist A, Liu G, Mirsaliotis A, and Xirodimas DP. Regulation of nucleolar signalling to p53 through NEDDylation of L11. *EMBO Rep.* 2009;10(10):1132-9.
88. Zhang J, Bai D, Ma X, Guan J, and Zheng X. hCINAP is a novel regulator of ribosomal protein-HDM2-p53 pathway by controlling NEDDylation of ribosomal protein S14. *Oncogene.* 2014;33(2):246-54.
89. Xiong X, Cui D, Bi Y, Sun Y, and Zhao Y. Neddylation modification of ribosomal protein RPS27L or RPS27 by MDM2 or NEDP1 regulates cancer cell survival. *Faseb j.* 2020;34(10):13419-29.
90. Watson IR, Blanch A, Lin DC, Ohh M, and Irwin MS. Mdm2-mediated NEDD8 modification of TAp73 regulates its transactivation function. *J Biol Chem.* 2006;281(45):34096-103.
91. Maghames CM, Lobato-Gil S, Perrin A, Trauchessec H, Rodriguez MS, Urbach S, et al. NEDDylation promotes nuclear protein aggregation and protects the Ubiquitin Proteasome System upon proteotoxic stress. *Nat Commun.* 2018;9(1):4376.
92. Li H, Zhu H, Liu Y, He F, Xie P, and Zhang L. Itch promotes the neddylation of JunB and regulates JunB-dependent transcription. *Cell Signal.* 2016;28(9):1186-95.
93. Guan J, and Zheng X. NEDDylation regulates RAD18 ubiquitination and localization in response to oxidative DNA damage. *Biochem Biophys Res Commun.* 2019;508(4):1240-4.
94. Peng Z, Fang W, Wu B, He M, Li S, Wei J, et al. Targeting Smurf1 to block PDK1-Akt signaling in KRAS-mutated colorectal cancer. *Nat Chem Biol.* 2025;21(1):59-70.
95. Du MG, Liu F, Chang Y, Tong S, Liu W, Chen YJ, et al. Neddylation modification of the U3 snoRNA-binding protein RRP9 by Smurf1 promotes tumorigenesis. *J Biol Chem.* 2021;297(5):101307.
96. Li S, Dong Y, Wang X, Feng D, Luan T, Wang Z, et al. The neddylation of the RNA-dependent RNA polymerase 3D of Coxsackievirus B3 promotes viral replication. *J Virol.* 2025;99(11):e0153525.
97. Gu WJ, Liu XX, Shen YW, Gong YT, Chen YL, Lin J, et al. TRIM4 enhances small-molecule-induced neddylated-degradation of CORO1A for triple negative breast cancer therapy. *Theranostics.* 2024;14(18):7023-41.
98. Noguchi K, Okumura F, Takahashi N, Kataoka A, Kamiyama T, Todo S, et al. TRIM40

promotes neddylation of IKKγ and is downregulated in gastrointestinal cancers. *Carcinogenesis.* 2011;32(7):995-1004.
99. Rabut G, Le Dez G, Verma R, Makhnevych T, Knebel A, Kurz T, et al. The TFIIH subunit Tfb3 regulates cullin neddylation. *Mol Cell.* 2011;43(3):488-95.
100. Wang H, Zhong M, Cui B, Yan H, Wu S, Wang K, et al. Neddylation of Enterovirus 71 VP2 Protein Reduces Its Stability and Restricts Viral Replication. *Journal of virology.* 2022;96(10):e0059822.
101. Schulze-Niemand E, and Naumann M. The COP9 signalosome: A versatile regulatory hub of Cullin-RING ligases. *Trends Biochem Sci.* 2023;48(1):82-95.
102. Mendoza HM, Shen LN, Botting C, Lewis A, Chen J, Ink B, et al. NEDP1, a highly conserved cysteine protease that deNEDDylates Cullins. *J Biol Chem.* 2003;278(28):25637-43.
103. Park JB, Moon GH, Cho A, Kwon M, Park JW, Yi EC, et al. Neddylation of insulin receptor substrate acts as a bona fide regulator of insulin signaling and its implications for cancer cell migration. *Cancer Gene Ther.* 2024.
104. Watson IR, Li BK, Roche O, Blanch A, Ohh M, and Irwin MS. Chemotherapy induces NEDP1-mediated destabilization of MDM2. *Oncogene.* 2010;29(2):297-304.
105. Ferro A, Carvalho AL, Teixeira-Castro A, Almeida C, Tomé RJ, Cortes L, et al. NEDD8: a new ataxin-3 interactor. *Biochim Biophys Acta.* 2007;1773(11):1619-27.
106. Gong L, Kamitani T, Millas S, and Yeh ET. Identification of a novel isopeptidase with dual specificity for ubiquitin- and NEDD8-conjugated proteins. *J Biol Chem.* 2000;275(19):14212-6.
107. Hemelaar J, Borodovsky A, Kessler BM, Reverter D, Cook J, Kolli N, et al. Specific and covalent targeting of conjugating and deconjugating enzymes of ubiquitin-like proteins. *Mol Cell Biol.* 2004;24(1):84-95.
108. Wada H, Kito K, Caskey LS, Yeh ET, and Kamitani T. Cleavage of the C-terminus of NEDD8 by UCH-L3. *Biochem Biophys Res Commun.* 1998;251(3):688-92.
109. Artavanis-Tsakonas K, Misaghi S, Comeaux CA, Catic A, Spooner E, Duraisingh MT, et al. Identification by functional proteomics of a deubiquitinating/deNeddylating enzyme in Plasmodium falciparum. *Mol Microbiol.* 2006;61(5):1187-95.
